# The complete plastid genome of *Suaeda malacosperma* (Amaranthaceae/Chenopodiaceae), a vulnerable halophyte in coastal regions of Korea and Japan

**DOI:** 10.1080/23802359.2018.1437822

**Published:** 2018-03-21

**Authors:** Jong-Soo Park, In-Su Choi, Dong-Hyuk Lee, Byoung-Hee Choi

**Affiliations:** a Department of Biological Sciences, Inha University, Incheon, Republic of Korea;; b Plant Survey and Conservation Team, Baekdu-daegan National Arboretum, Bonghwa, Republic of Korea

**Keywords:** *Suaeda malacosperma*, plastid genome, halophyte

## Abstract

*Suaeda malacosperma* has limited distribution in the coastal regions of Korea and Japan and is named as a vulnerable halophyte in the Red List of Japan. The complete plastid genome of *S. malacosperma* is 151,989 bp long, and is composed of large single-copy (83,492 bp) and small single-copy (18,121 bp) regions plus two inverted repeats (25,188 bp each). The plastid genome encodes 130 genes, including 8 rRNAs, 37 tRNAs, and 83 protein-coding genes. *rpl23* is pseudogenized. Phylogenetic analysis showed a sister relationship between *Suaeda* and *Bienertia.* This complete plastid genome is the first reported in genus *Suaeda*.

*Suaeda malacosperma* Hara is annual halophyte for which distribution is restricted to salt marshes in Korea and Japan (Shim et al. 2001). In Korea, it was recently reported as an unrecorded species, occurring only in brackish tidal marshes that can easily be disturbed (Shim et al. 2001). This species has been assigned Endangered Rank II (VU) in the Red List of Japan (https://ikilog.biodic.go.jp/Rdb/booklist). Wide swaths of habitat for halophytes in tidal regions are disappearing due to land reclamation and other human activities (Suzuki 2003; Millennium Ecosystem Assessment 2005; Choi 2014). Several local populations in Japan are now considered extinct (Nakanishi 2001). The complete plastid genome of *S. malacosperma* is the first report for any member of the genus *Suaeda*, and these details provide fundamental genetic information that could be used in developing genetic markers for conservation and in devising a phylogenomics approach for phylogenetic studies.

Total genomic DNA was extracted from silica-gel dried leaves of a single individual collected at Boseong, Korea (34°50′N, 127°24′E). The voucher specimen (J.S. Park 1610231) was deposited at the herbarium of Korea National Arboretum (KH). Genomic DNA was sequenced using the Illumina Miseq platform (LAS, Seoul, Korea). To assemble the plastid genome sequence, we generally followed the procedure of Wang and Messing (2011), but with minor modifications (Choi and Choi 2017). The plastid genome of *Bienertia sinuspersici* Akhani (GenBank: KU726550) (Kim et al. 2016) served as our reference genome. This draft genome was annotated using DOGMA (Wyman et al. 2004), tRNAscan-SE (Lowe and Chan 2016), and the plastid genomes of *B. sinuspersici*, *Haloxylon ammodendron* (C.A. Mey.) Bunge (GenBank: KF534478), and *H. persicum* Bunge ex Boiss. & Buhse (GenBank: KF534479).

The plastid genome of *S*. *malacosperma* (GenBank: MG813535) is 151,989 bp long and comprises a large single-copy region (LSC; 83,492 bp), a small single-copy region (SSC; 18,121 bp), and a pair of inverted repeats (IRs; 25,188 bp) that are separated by the LSC and SSC. This genome encodes 130 genes, including 8 rRNAs, 37 tRNAs, and 83 protein-coding genes. Among these, 16 genes are duplicated in the IR region. While 17 genes contain a single intron, four others have two introns each. One gene, *rpl23*, is pseudogenized and known in *Haloxylon* (Dong et al. 2016). The overall GC content for *S*. *malacosperma* is 36.4%.

To construct a maximum likelihood (ML) phylogenetic tree, we extracted 69 genes from the genome sequences for each of 12 species within Chenopodiaceae and included *Amaranthus tricolor* L. of Amaranthaceae as the outgroup. The sequences were aligned using MAFFT v.7.309 (Katoh and Standley 2013) and the tree was analyzed with RAxML 8.2.11 (Stamatakis 2014) (Figure 1). The topology of this ML tree for 69 genes from the plastid genomes of Chenopodiaceae members largely corresponded with that produced from a previous study based on *rbcL* (Kadereit et al. 2003). *S*. *malacosperma* was sister to *B. sinuspersici*.

**Figure 1.**
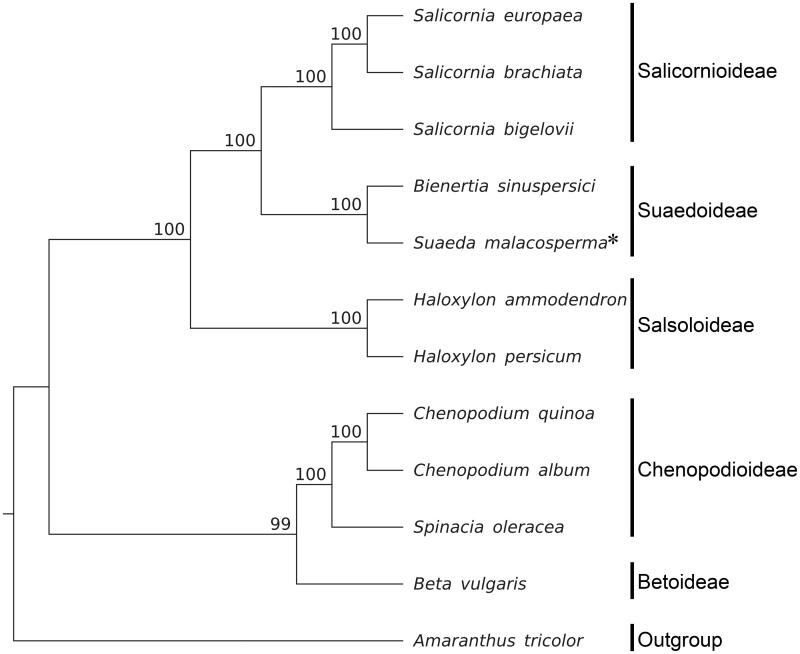
Molecular phylogeny of Chenopodiaceae using 69 genes from plastid genomes of 12 species including 1 species from Amaranthaceae as outgroup. Bootstrap values are based on 1000 replicates; values are shown near each node. Plastid genome accession number is used in this phylogeny analysis: *Salicornia europaea*, KJ629116; *S. brachiata*, KJ629115; *S. bigelovii*, KJ629117; *B. sinuspersici*, KU726550; *H. ammodendron*, KF534478; *H. persicum*, KF534479; *Chenopodium quinoa*, KY419706; *C. album*, KY419707; *Spinacia oleracea*, AJ400848; *Beta vulgaris*, KR230391; *Amaranthus tricolor*, KX094399.
